# Exercise intervention to reduce mobile phone addiction in adolescents: a systematic review and meta-analysis of randomized controlled trials

**DOI:** 10.3389/fpsyg.2023.1294116

**Published:** 2023-12-13

**Authors:** Zuxian Li, Xue Xia, Qilong Sun, Yansong Li

**Affiliations:** ^1^School of Physical Education, Qingdao University, Qingdao, China; ^2^School of Social Development and Health Management, University of Health and Rehabilitation Sciences, Qingdao, China; ^3^Liaocheng Infant Normal School, Liaocheng, China

**Keywords:** exercise, mobile phone addiction, adolescent, randomized controlled trial, meta-analysis

## Abstract

**Background:**

The growing problem of adolescent mobile phone addiction has attracted significant attention, underscoring the importance of identifying approaches to address it. Exercise has been found to reduce adolescent mobile phone addiction; however, its mechanism remains unclear. This review aims to elucidate the potential moderating factors between exercise and mobile phone addiction based on previous studies to provide a reference for adolescents to effectively participate in exercise to reduce mobile phone addiction.

**Methods:**

Articles were searched in the CNKI, Web of Science, Scopus, Cochrane, PsycINFO, and PubMed databases according to the inclusion criteria and followed the Preferred Reporting Items for Systematic Assessment and Meta-analysis (PRISMA). The quality of the literature was assessed by two independent reviewers using the Cochrane Collaboration Risk of Bias tool for methodological quality assessment. Meta-analysis was performed using Stata 15.1 software for Meta-analysis, standardized mean difference (SMD) was combined using a random effects model, and subgroup analysis was used to explore heterogeneity.

**Results:**

A total of 12 studies, 17 samples, and 861 subjects were included in the meta-analysis, and all studies were randomized controlled trials. The findings revealed that the exercise intervention significantly reduced mobile phone addiction in adolescents (SMD = −3.11; 95% CI: −3.91, −2.30; *p* < 0.001). The intervention effect was moderated by multiple variables, such as the measurement tools, exercise intervention types, cycles, frequency, and duration of a single exercise intervention.

**Conclusion:**

Our findings suggest that exercise could serve as an effective strategy for preventing or ameliorating mobile phone addiction in adolescents. Based on the results of this study, we encourage mobile phone-addicted adolescents to engage in a single exercise using a mix of skills for 30–60 min three or more times weekly for more than eight consecutive weeks.

## 1 Introduction

With continuously developing information technology, mobile phones have become increasingly popular. Recently, mobile phones have become an important part of daily life worldwide (Chen et al., 2017). Relevant data indicate that mobile phone users worldwide are growing exponentially and are expected to reach 7.516 billion by 2026 (Croce et al., 2021). While mobile phones bring convenience, they pose potential risks, leading to new behavioral problems, including mobile phone addiction (Ezoe et al., 2009).

Mobile phone addiction is also called “mobile phone syndrome,” “mobile phone dependence,” “mobile phone addiction syndrome,” and “mobile phone anxiety disorder” (Zhang et al., 2021). Mobile phone addiction is considered an impulse control disorder, which is defined as inappropriate use of a mobile phone or excessive use of a mobile phone, resulting in a loss of control over the use of a mobile phone (Liu and Lu, 2022), disrupting daily life, and causing extreme emotional changes and severe physical reactions in the individual (Sert et al., 2019), manifested as physical health problems in mobile phone users, including mobile phone addicts, such as headaches, blurred vision, memory loss, and hearing impairment (Dol, 2016). Sedentary behaviors and participation in specific sedentary activities (e.g., screen-based behaviors) have rapidly emerged as potential additional risk factors for adolescent health and well-being (van Sluijs et al., 2021). Developing an incorrect posture with the head tilted forward due to prolonged mobile phone use may lead to problems and pain in the cervical spine and shoulders (Warda et al., 2023). Previous studies have found a strong relationship between mobile phone addiction and musculoskeletal complications, leading to various musculoskeletal problems due to sedentary and prolonged static standing, which can cause muscle pain (Toh et al., 2017; Xie et al., 2017; Zirek et al., 2020; Mustafaoglu et al., 2021) and affect work efficiency and personal quality of life. Mobile phone addiction can also cause several mental disorders, including stress, insomnia, and general negative emotions, and prolonged mobile phone use may also trigger potential conditions such as decreased sleep quality and neurasthenia (Tamura et al., 2017; Višnjić et al., 2018). Adolescents in the rapid mental and cognitive development stage have a higher risk of mobile phone addiction than other age groups (Lee and Lee, 2017) due to the lack of self-control, a strong desire to seek the use of new technology, and other characteristics (Long et al., 2016; Li et al., 2018). Mobile phone addiction has become an emerging issue that threatens the academic, physical, and mental health of adolescents (Mok et al., 2014); it is more pervasive and insidious than traditional Internet addiction (Beranuy et al., 2009). Studies have demonstrated that failure to timely reduce adolescent mobile phone addiction may lead to an increasing frequency of various mood disorder problems. Hence, examining ways to effectively reduce mobile phone addiction is urgent.

The idea that exercise is beneficial for physical and mental health has recently become well known (Kuan et al., 2019; Anderson and Fowers, 2020; Li et al., 2021) and has been recognized worldwide to provide many important benefits to individuals, including but not limited to a healthier lifestyle, improved cardiorespiratory fitness, and increased muscle strength and life expectancy (Janssen et al., 2013; Rebar et al., 2015). It can also effectively reduce mental health issues, such as anxiety and depression (LeBouthillier and Asmundson, 2017). To the best of our knowledge, there have been limited systematic reviews (Liu et al., 2022; Xiao et al., 2022; Zhang et al., 2023) showing the association between exercise and mobile phone addiction, but the effect of exercise on reducing mobile phone addiction in adolescents requires further exploration. Our goal was to systematically assess the impact of exercise on reducing mobile phone addiction in adolescents through a meta-analysis, clarifying potential moderating effects and offering guidance for effective exercise participation.

## 2 Methods

This meta-analysis followed the Preferred Reporting Items for Systematic Reviews and Meta-Analyses (PRISMA; Page et al., 2021). Because all data were extracted from previously published studies, no patient consent or ethical approval was required. This study was conducted as follows: (i) defining inclusion and exclusion criteria, (ii) literature search, (iii) selection of studies, (iv) data extraction, (v) assessment of publication bias and quality of studies, and (vi) data analysis.

### 2.1 Inclusion and exclusion criteria

We used the PICOS strategy to define the sample characteristics and profiles of the included studies.

Inclusion criteria:

1. Tested and diagnosed as adolescents with mobile phone dependence.
2. Physical health without comorbidities with other types of illnesses.
3. No previous participation in a similar research program (use of medication for psychiatric problems or other psychotherapies).
4. The experimental group was an exercise intervention that did not combine other interventions (e.g., cognitive training).
5. Conclusion indicators included mobile phone addiction scores, and statistics included mean and standard deviation.
6. The study design is a randomized controlled trial (RCT).
7. Employed a pre−/post-test framework, including experimental and control group samples, for pre-test and post-test values of mobile phone addiction scores.

Exclusion criteria:

1. Duplicate studies.
2. Literature review combined with meta-analysis.
3. Descriptive studies, case–control studies, qualitative reports, and case reports.
4. Non-journal papers, such as dissertations.
5. Articles with abstracts only and no full text.
6. Non-randomized controlled study design.
7. Studies without a control group.
8. Literature in which the questionnaire instrument lacks reliability and validity.
9. Literature with incomplete information and data on outcome indicators, resulting in data not being extracted.
10. Non-English or non-Chinese literature.

### 2.2 Literature search

We searched six electronic databases (CNKI, Web of Science, Scopus, Cochrane, PsycINFO, and PubMed) for RCTs on the effects of exercise interventions on mobile phone addiction from the inception of the databases to the present day, with English and Chinese as the only acceptable languages. The specific search strategy was as follows: (“cell phone” OR “cellular phone” OR “cellular telephone” OR “mobile devices” OR “mobile phone” OR “smart phone” OR “smartphone” OR “touch screen phone” OR “hand-held device”) AND (“addiction” OR “dependence” OR “dependency” OR “abuse” OR “addicted to” OR “overuse” OR “problem use” OR “compensatory use” OR “smartphone use disorder” OR “problematic smartphone use” OR “problematic smart phone use” OR “problematic mobile phone use” OR “problematic cell phone use” OR “problematic cellular phone use” OR “Nomophobia”) AND (“exercise” OR “exercises” OR “physical activity” OR “activities, physical” OR “activity, physical” OR “physical activities” OR “exercise, physical” OR “exercises, physical” OR “physical exercise” OR “physical exercises” OR “acute exercise” OR “acute exercises” OR “exercise, acute” OR “exercises, acute” OR “exercise, isometric” OR “exercises, isometric” OR “isometric exercises” OR “isometric exercise” OR “exercise, aerobic” OR “aerobic exercise” OR “aerobic exercises” OR “exercises, aerobic” OR “exercise training” OR “exercise trainings” OR “training, exercise” OR “trainings, exercise”). References of the studies were also retrospectively included to supplement the relevant literature (Table 1).

**Table 1:** Search subject headings for this study.

Group	Algorithm
Group 1	“cell phone” OR “cellular phone” OR “cellular telephone” OR “mobile devices” OR “mobile phone” OR “smart phone” OR “smartphone” OR “touch screen phone” OR “hand-held device”
Group 2	“addiction” OR “dependence” OR “dependency” or “abuse” OR “addicted to” OR “overuse” OR “problem use” OR “compensatory use” OR “smartphone use disorder” OR “problematic smartphone use” OR “problematic smart phone use” OR “problematic mobile phone use” OR “problematic cell phone use” OR “problematic cellular phone use” OR “Nomophobia”
Group 3	“exercise” OR “exercises” OR “physical activity” OR “activities, physical” OR “activity, physical” OR “physical activities” OR “exercise, physical” OR “exercises, physical” OR “physical exercise” OR “physical exercises” OR “acute exercise” OR “acute exercises” OR “exercise, acute” OR “exercises, acute” OR “exercise, isometric” OR “exercises, isometric” OR “isometric exercises” OR “isometric exercise” OR “exercise, aerobic” OR “aerobic exercise” OR “aerobic exercises” OR “exercises, aerobic” OR “exercise training” OR “exercise trainings” OR “training, exercise” OR “trainings, exercise”

### 2.3 Study selection and data collection process

We designed a data form that both authors used to extract data from eligible studies, compare the extracted data, and discuss the resolution of all discrepancies.

Relevant information extracted from the studies included the following:

1. Basic information: first author and year of publication.
2. Participant demographics: age, sample size (total number and number of people in each group and their attrition rate), and information on the control group.
3. Interventions and comparisons: mobile phone addiction assessment tool, type of intervention, and details of the intervention program (duration, frequency, and total intervention cycle).
4. Outcomes: scores on the mobile phone addiction scale, means and standard deviations, and variables used for subgroup analysis. If these data were missing in the article or additional files, the relevant author was contacted to obtain them.

The considered studies were added to the Endnote X7 reference management software to remove duplicates. Two researchers (i.e., ZL and QS) individually reviewed the studies initially added based on the inclusion criteria, including the first author, publication year, participant age, sample size, outcomes related to the mobile phone addiction scale scores, variables used for subgroup analyses, and control group information. If these data were missing in the article or additional files, the relevant author was contacted to obtain them. When two researchers disagreed on the extracted information, a third author (XX) reassessed it to reach a consensus.

### 2.4 Data extraction and outcome measures

The researchers searched the literature sequentially, imported the retrieved literature into Endnote X7 software, removed duplicates, and the remaining literature was screened according to the inclusion and exclusion criteria by reading the titles, abstracts, and full text for re-screening, and extracted the literature that met the criteria. After data screening and extraction, two researchers cross-checked the analysis process, and the final selected articles were de-topped by the two researchers in agreement. The predefined standardized format of population, intervention, control, and outcome (PICO) was followed (Moyer, 2008).

The main information extracted for inclusion in the literature included the following:

1. General information: authors of the study, title, year of publication, etc.
2. Characteristics relevant for inclusion in the analysis: sample size, study population, exercise intervention types, cycles, frequency, duration of a single exercise intervention, intensity of exercise, and number of people in the experimental and control groups.
3. Outcome metrics and data: measurement tools (questionnaire scales), means, standard deviations, *p*-values, etc.

### 2.5 Risk of bias

Data were organized using Microsoft Excel 2019 software. Two investigators independently conducted a literature risk of bias assessment for studies screened for inclusion using the Cochrane Handbook for Assessing Risk of Bias. In case of disagreement, a third investigator was conferred until the opinions were harmonized. The following seven main areas were assessed: randomized grouping methods, allocation concealment, blinded implementation of subjects and investigators, blinded implementation of outcome assessment, completeness of outcome data, selection of reports, and other biases to evaluate the risk of bias of included studies (Cumpston et al., 2019). Based on the seven dimensions addressed by the tool and the description of each piece of literature, the researcher evaluated low risk of bias, uncertain risk of bias, and high risk of bias. Any disagreements were resolved through researcher discussion and consensus.

### 2.6 Statistical analyses

We performed a standard paired meta-analysis and extracted all data from the included studies. To include as many eligible studies as possible, the mean and standard deviation (SD) of the baseline and endpoints were first extracted. If data were missing, an attempt was made to convert the data into a standard format.

Due to measurement errors of outcome indicators, standardized mean differences (SMD) were chosen as effect indicators to combine the effect sizes (Zhang et al., 2022), with each effect size expressed as a 95% confidence interval (CI). For the SMD interpretation, we used the following benchmark anchors: 0.2 for small effect sizes, 0.5 for medium effect sizes, and 0.8 for large effect sizes (Cohen, 1992). Egger tests were used to analyze publication bias. The $I^{2}$ statistic was used to measure heterogeneity between studies. An $I^{2}$ -value of 0% suggested no heterogeneity, while values of 25, 50, and 75% indicated low, moderate, and high heterogeneity, respectively; significant heterogeneity was considered to exist if $I^{2}$ > 50% (Higgins, 2003). If significant heterogeneity existed, the source was further examined using subgroup analysis. If heterogeneity was statistically insignificant, the fixed-effects model was selected for meta-analysis; conversely, the random-effects model was selected; the heterogeneity sources arising from it were explored without affecting the integrity of the analysis results, and subgroup analysis was selected to assess the heterogeneity sources according to the classification of factors. To better detect heterogeneity in different variables and sources of variability in the results, subgroup analyses were conducted on the main outcomes, focusing on variables including mobile phone addiction measurement tools, exercise intervention types, cycles, frequency, and duration of a single exercise intervention. Heterogeneity sources were further examined using subgroup and sensitivity analyses. Differences were considered statistically significant at *p* < 0.05. All analyses used Stata 15.1 (Stata, Corp, College Station, Texas, United States).

## 3 Results

### 3.1 Search results

**Figure 1:**
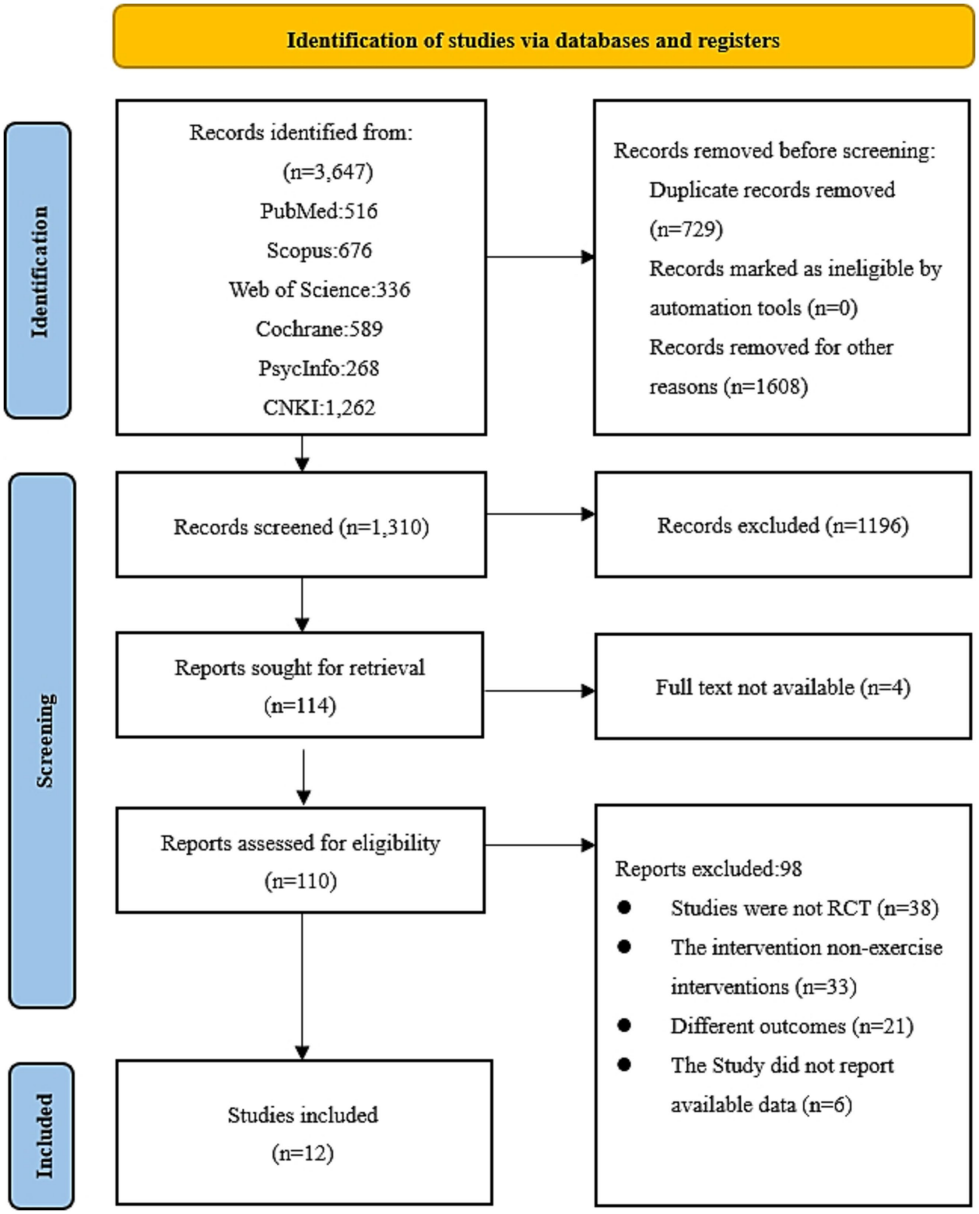
PRISMA flow diagram of the paper selection process used in the present study.

We retrieved 3,647 studies from databases, of which 729 duplicates were first excluded, 1,608 were excluded after reading the article title, and 1,196 were removed based on the abstract. Subsequently, 114 studies were eligible for full-text screening, and 102 papers were excluded for the following reasons: 4 studies were unavailable in full text, 38 were not RCT studies, 33 had interventions that were not exercise interventions, 21 were inconsistent with our outcome metrics, and 6 studies did not present usable data for our analyses. The literature screening process is illustrated in Figure 1.

### 3.2 Characteristics of included studies

This review included 12 studies published between 2014–2023 (Bu, 2014; Ge et al., 2015; Wang, 2016; Zhu, 2017; Chen, 2020; Lu et al., 2020; Fan, 2021; Wang, 2021; Xiao et al., 2021; Liao, 2022; Liu, 2022; Zhang, 2022; Table 2). Ten studies were conducted in Chinese and two in English. A total of 17 independent samples were reported, with a total sample size of 861 individuals (maximum sample size of 65 and minimum sample size of 16), including 421 in the experimental group and 440 in the control group. The extracted literature information was coded according to eigenvalues to facilitate the meta-analysis.

**Table 2:** Key characteristics of the studies reviewed (authors, sample size, intervention instrument, measurement instrument, intervention period, frequency of intervention, and duration of individual interventions.

Authors (year)	N		Measurement tools	Types of exercise interventions	Cycles of exercise interventions	Frequency of intervention	Duration of a single intervention
	Experimental	Control					
Bu (2014)	30	30	MPATS	Mixed skills	24 Weeks	3–5 times/week	Not reported
Chen (2020)	25	25	MPATS	Mixed skills	8 Weeks	3 times per week	More than 60 min
Zhang (2022)	17	20	MPATS	Mixed skills	8 Weeks	2 times per week	60 min
Zhu (2017)	30	30	MPATS	Mixed skills	8 Weeks	3 times per week	40 min
Wang (2021)	30	30	MPATS	Mixed skills	18 Weeks	2 times per week	Not reported
Lu et al.(2020)	31	34	MPAI	Closed skills	12 Weeks	2 times per week	70 min
Ge et al.(2015)	18	18	MPAI	Open skills	18 Weeks	3 times per week	Not reported
Xiao et al. (2021)	31	34	MPAI	Open skills	12 Weeks	3 times per week	70 min
Xiao et al. (2021)	31	34	MPAI	Closed skills	12 Weeks	3 times per week	70 min
Liao (2022)	8	8	MPAI	Open skills	6 Weeks	Not reported	Not reported
Liao (2022)	8	8	MPAI	Closed skills	6 Weeks	Not reported	Not reported
Liu (2022)	31	34	MPAI	Open skills	10 Weeks	2 times per week	60 min
Liu (2022)	31	34	MPAI	Closed skills	10 Weeks	2 times per week	60 min
Fan (2021)	32	32	SAS-C	Mixed skills	14 Weeks	3 times per week	60 min
Fan (2021)	32	32	SAS-C	Mixed skills	14 Weeks	3 times per week	60 min
Wang (2016)	11	12	SAS-C	Open skills	7 Weeks	3 times per week	45 min
Wang (2016)	25	25	SAS-C	Open skills	12 Weeks	3 times per week	45 min

### 3.3 Quality of the included studies

**Figure 2:**
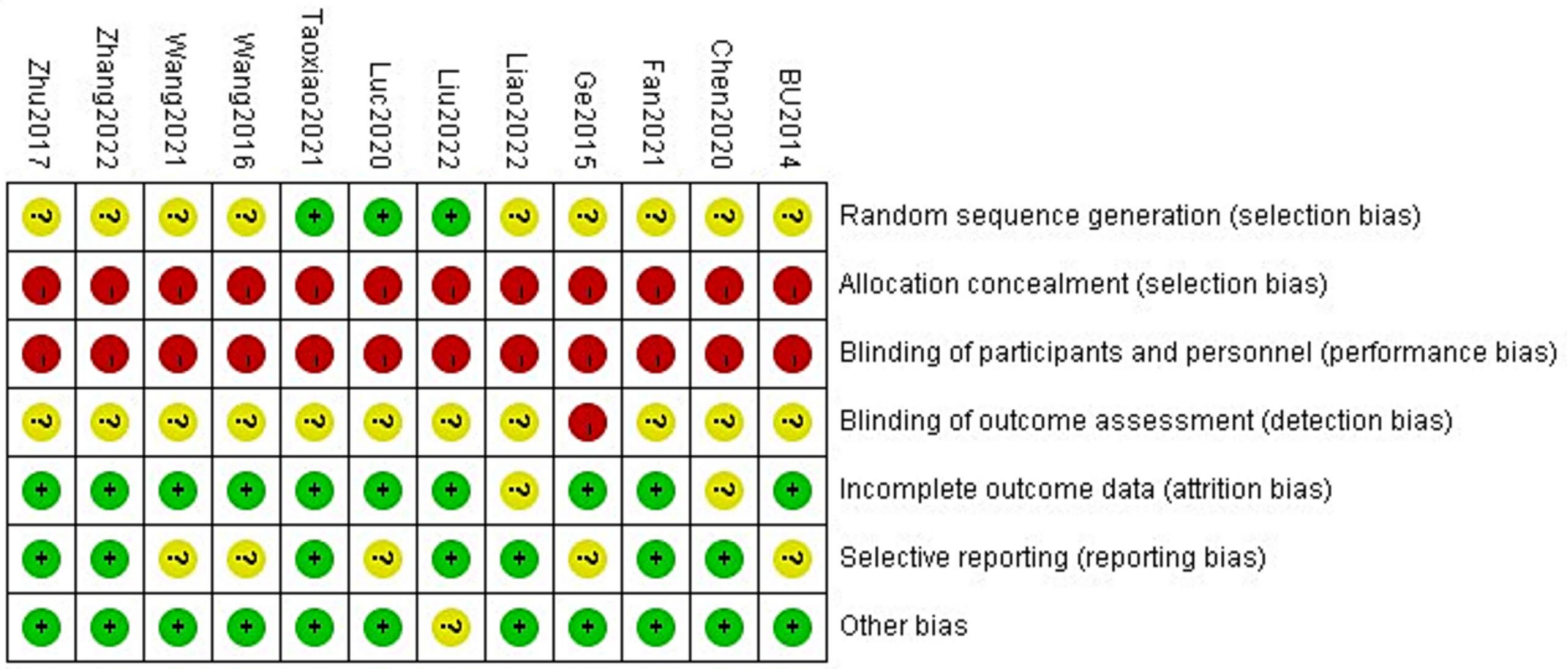
Assessment of the methodological quality of the included literature.

Three of the 12 included studies had a low risk of bias in randomized sequence generation, none had a low risk of bias in allocation concealment or implementation, and one had a high risk of bias in measurement. Ten studies had a low risk of bias when assessing follow-up bias, seven had a low risk of bias when assessing reporting bias, and 11 had a low risk of bias when assessing other biases. Detailed information is displayed in Figure 2.

### 3.4 Analysis of the overall effect of exercise on mobile phone addiction intervention for adolescents

**Figure 3:**
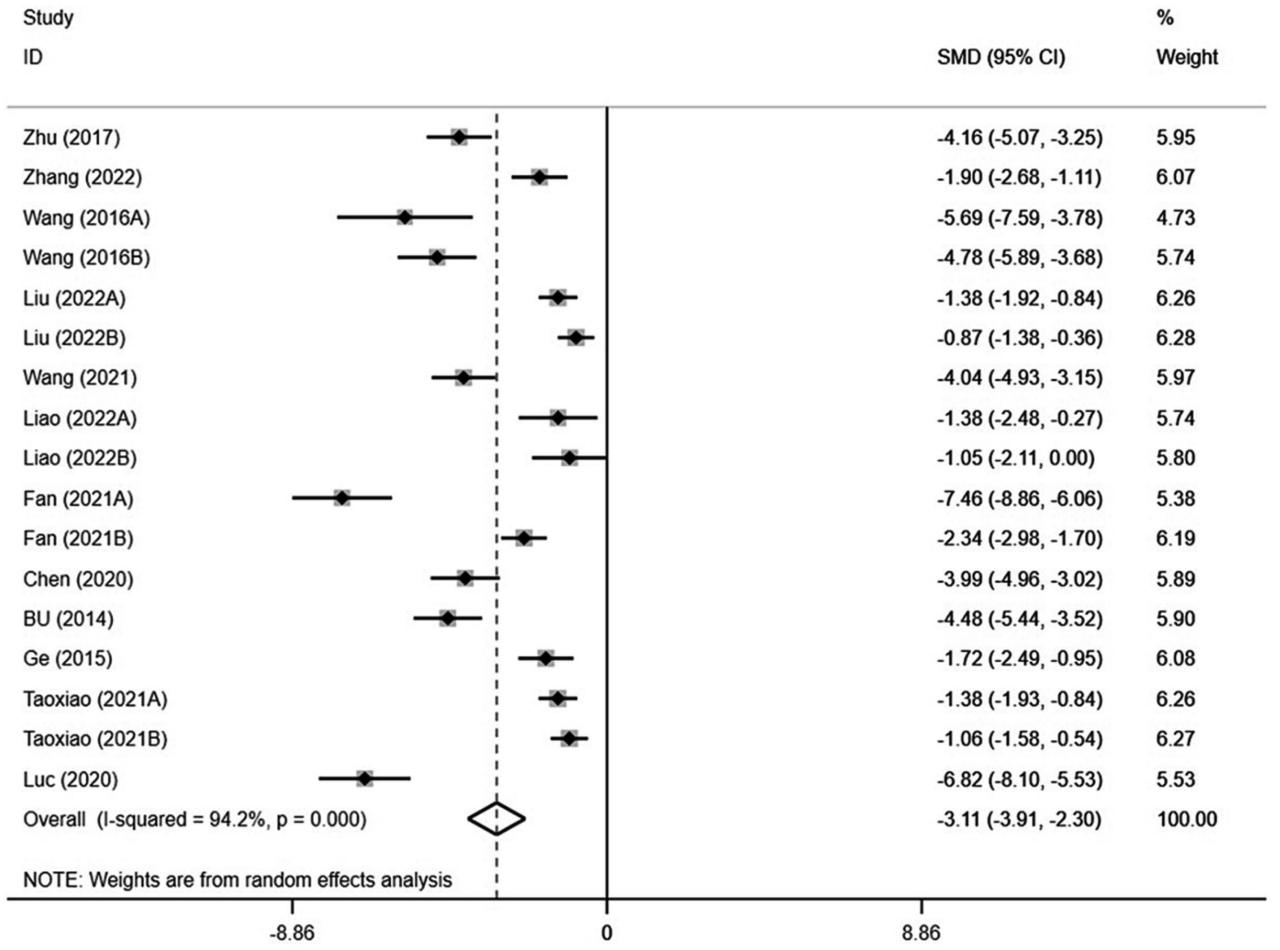
Forest plot showing standardized mean difference (95% CI).

A total of 12 studies containing 17 independent exercise intervention samples with 861 subjects were included (Table 2). Tests for heterogeneity displayed significant heterogeneity between studies (Q = 277.16; *p* < 0.001; $I^{2}$ = 94.2%); therefore, a meta-analysis was performed using a random-effects model (Table 3). The results depicted that the combined effect size of the exercise interventions was SMD = −3.11, 95% CI: −3.91 to −2.30 and was statistically significant (*p* < 0.001). The absolute value of the combined effect size was higher than 0.80 according to the COHEN evaluation criteria, indicating that the exercise interventions significantly improved mobile phone addiction in adolescents (Figure 3). The $I^{2}$ = 94.2% > 50%, indicating strong heterogeneity and heterogeneity source needs further exploration.

**Table 3:** Analysis of the overall effect of exercise on the effect of mobile phone addiction intervention for adolescents.

	K	N	SMD	95% CI	Homogeneity test			Test of null (two-tailed)	
					Q	*p*	$I^{2}$	Z-value	*p*
Random effects model	17	861	−3.11	(−3.91, −2.30)	277.16	0.000	94.2%	7.59	0.000

#### 3.4.1 Results of the test for moderating effects

As there was heterogeneity between studies in the overall effects test, to explore the heterogeneity sources, this study conducted moderated tests to assess the effects of the five moderating variables on the role of exercise interventions in improving adolescent mobile phone addiction (Table 4).

**Table 4:** Examining the moderating effect of exercise on the effect of mobile phone addiction intervention in adolescents.

Adjustment variables	K	N	SMD	95% CI	Test of null (two-tailed)	
					Z-value	*p*
Measurement tools						
MPATS	5	267	−3.69	[−4.68, −2.70]	7.30	< 0.001
MPAI	8	393	−1.84	[−2.64, −1.04]	4.51	< 0.001
SAS-C	4	201	−5.01	[−7.42, −2.59]	4.07	< 0.001
Types of exercise interventions						
Open skills	6	255	−2.51	[−3.57, −1.45]	4.65	< 0.001
Closed skills	4	211	−2.36	[−4.18, −0.55]	2.55	0.011
Mixed skills	7	395	−3.98	[−5.11, −2.84]	6.88	0.011
Cycles of exercise interventions						
≤ 8 weeks	6	243	−2.48	[−3.44, −1.52]	5.06	< 0.001
> 8 weeks	11	618	−3.49	[−4.61, −2.36]	6.08	< 0.001
Frequency of exercise interventions						
2 times a week	5	292	−2.92	[−4.51, −1.33]	3.60	< 0.001
3 times per week	10	537	−3.60	[−4.70, −2.49]	6.37	< 0.001
Duration of single exercise interventions						
30–60 min	8	428	−3.44	[−4.70, −2.17]	5.33	< 0.001
> 60 min	4	245	−3.23	[−5.20, −1.26]	3.21	< 0.001

#### 3.4.2 The mobile phone addiction measurement tools

Twelve studies (Bu, 2014; Ge et al., 2015; Wang, 2016; Zhu, 2017; Chen, 2020; Lu et al., 2020; Fan, 2021; Wang, 2021; Xiao et al., 2021; Liao, 2022; Liu, 2022; Zhang, 2022), containing a total of 861 cases, were included, and three groups were highly heterogeneous regarding effect size differences ( $I^{2}$ = 94.2%), indicating a significant moderating effect of the mobile phone addiction measurement tools on intervention effects. The effect sizes for the mobile phone addiction measurement tools were MPATS: SMD = −3.69, 95% CI: [−4.68, −2.70]; MPAI: SMD = −1.84, 95% CI: [−2.64, −1.04]; and SAS-C: SMD = −5.01, 95% CI: [−7.42, −2.59], all of which were statistically significant (*p* < 0.001).

#### 3.4.3 Types of exercise interventions

Twelve studies (Bu, 2014; Ge et al., 2015; Wang, 2016; Zhu, 2017; Chen, 2020; Lu et al., 2020; Fan, 2021; Wang, 2021; Xiao et al., 2021; Liao, 2022; Liu, 2022; Zhang, 2022) containing a total of 861 cases were included. There was high heterogeneity in the effect sizes ( $I^{2}$ = 94.2%) across the three groups. The effect sizes for the types of exercise intervention were open skills: SMD = −2.51, 95% CI: [−3.57, −1.45], *p* < 0.001; closed skills: SMD = −2.36, 95% CI: [−4.18, −0.55], *p* < 0.001; and mixed skills: SMD = −3.98, 95% CI: [−5.11, −2.84], *p* < 0.001. Mixed skills had the largest effect size.

#### 3.4.4 Cycles of exercise interventions

Twelve studies (Bu, 2014; Ge et al., 2015; Wang, 2016; Zhu, 2017; Chen, 2020; Lu et al., 2020; Fan, 2021; Wang, 2021; Xiao et al., 2021; Liao, 2022; Liu, 2022; Zhang, 2022) containing a total of 861 cases were included. There was high heterogeneity in the effect size differences between the two groups ( $I^{2}$ = 94.2%), indicating a significant moderating effect of the intervention cycle on the intervention effect. The effect sizes for exercise intervention cycles were as follows: ≤ 8 weeks: SMD = −2.48, 95% CI: [−3.44, −1.52], *p* < 0.001; and > 8 weeks: SMD = −3.49, 95% CI: [−4.61, −2.36], *p* < 0.001, with the largest effect size for exercise intervention cycles above 8 weeks.

#### 3.4.5 Frequency of exercise interventions

Eleven studies (Bu, 2014; Ge et al., 2015; Wang, 2016; Zhu, 2017; Chen, 2020; Lu et al., 2020; Fan, 2021; Wang, 2021; Xiao et al., 2021; Liu, 2022; Zhang, 2022) containing 829 cases were included. There was high heterogeneity in the effect size differences between the two groups ( $I^{2}$ = 94.8%), indicating a significant moderating effect of the frequency of exercise intervention on the intervention effect. Of the 12 included studies, one did not explicitly report intervention frequency, and the remaining studies contained two intervention frequencies: two times/week, three times, and more/week. Two times/week: SMD = −2.92, 95% CI: [−4.51, −1.33], *p* < 0.001; three times and more/week: SMD = −3.60, 95% CI: [−4.70, −2.49], *p* < 0.001. This indicates that exercise interventions performed more than twice per week have a positive effect on reducing mobile phone addiction, whereas the frequency of interventions performed thrice or more weekly had the highest effect.

#### 3.4.6 Duration of single exercise interventions

Eight studies (Wang, 2016; Zhu, 2017; Chen, 2020; Lu et al., 2020; Fan, 2021; Wang, 2021; Xiao et al., 2021; Liu, 2022; Zhang, 2022) reported on the duration of single exercise interventions, containing 673 cases, were included. Two groups were highly heterogeneous regarding effect size differences ( $I^{2}$ = 95.2%), indicating a significant modifying effect of single-exercise intervention duration on intervention effects. The combined effect sizes for different single intervention durations were 30–60 min/session: SMD = −3.44, 95% CI: [−4.70, −2.17]; and > 60 min/session: SMD = −3.23, 95% CI: [−5.20, −1.26], *p* < 0.001. The included single-intervention durations were divided by the main exercise time, and 30–60 min had the largest intervention time effect size.

### 3.5 Risk of bias

We performed Egger’s regression to test whether the results were biased by the effect sizes of the different sources (Egger et al., 1997) and found a significant bias (*t* = −5.22, *p* < 0.001, Table 5).

**Table 5:** Egger test results.

Standard efficiency	Coefficient	Standard error	T	*P* > |t|	95% ci
*Slope*	1.368	0.726	1.88	0.079	(−0.179, 2.916)
*Bias*	−9.637	1.846	−5.22	0.000	(−13.571, −5.703)

## 4 Discussion

Mobile phones have become essential communication tools in modern society. Some studies have demonstrated a correlation between mobile phone use and age (Zagalaz-Sánchez et al., 2019), with university students and younger people using their phones more frequently in their daily lives. Mobile phones offer a means to address challenges and an avenue for detrimental escapism from reality. Adolescents are in a critical period of physical and mental development, and because they have less control and cannot plan their mobile phone use rationally, they can easily become addicted to their phones, further damaging their physical and mental health (Long et al., 2016).

Mobile phone addiction not only increases sedentary behavior but is also fueled by prolonged inactivity, suggesting a link between addiction and increased sedentary time (Yang et al., 2019). Some studies suggest that mobile phone-addicted students are more sedentary daily and have less weekly physical activity than non-mobile phone-addicted students (Kim et al., 2015). A cross-sectional study concluded that mobile phone addiction decreases physical fitness among adolescents, which supports the need for intervention in mobile phone addiction (Al-Amri et al., 2023).

Few studies have conducted meta-analyses on the effects of exercise on mobile phone addiction, and most of these are observational rather than RCTs. Our meta-analysis, based on RCTs, revealed that exercise significantly reduced adolescent mobile phone addiction. According to a related meta-analysis, exercise significantly reduces adolescent mobile phone addiction (Xiao et al., 2022), lending credence to the findings of this investigation. Engaging in exercise decreases sedentary time and moderates daily mobile phone use. Exercise also positively affects the mental health of adolescents, as it can improve their self-esteem and well-being in the real world, eliminate the need for adolescents to gain affirmation and self-satisfaction in the virtual world, and effectively reduce mobile phone addiction in adolescents. It has been proved that there is a significant negative correlation between the level of physical exercise and mobile phone addiction (Guo et al., 2022). This means that the higher the level of physical exercise, the lower the degree of mobile phone addiction. Therefore, more attention should be paid to the physical exercise of young people, and we should provide them with a good exercise environment to prevent them from becoming addicted to the Internet and mobile phones.

### 4.1 Discussion of results

With an overall heterogeneity of $I^{2}$ = 94.2% > 50% for exercise interventions to reduce adolescent mobile phone addiction, it can be assumed that the effect sizes of individual studies are discrete, and moderating variables should be introduced to investigate heterogeneity in greater detail.

#### 4.1.1 The moderating effects of measurement tools

Measurement tools are the primary focus of mobile phone addiction analyses. The Mobile Phone Problem Use Scale (MPPUS), developed in 2005 (Bianchi and Phillips, 2005), the Smartphone Addiction Scale (SAS), which was developed based on the Korean Internet Addiction Scale and the functional characteristics of smartphones, and the Smartphone Addiction Scale for Teenagers (SAS-SV) are notable instruments (Kwon et al., 2013). This review used the MPATS, MPAI, and SAS-C scales, characterized by robust reliability and validity testing. Furthermore, alongside these scales, numerous other tools exist to gauge mobile phone addiction. For example, a study that used a self-assessment questionnaire similarly concluded that a comprehensive exercise-based intervention had a more pronounced effect on alleviating the symptoms of mobile phone dependence in college students and improving their quality of sleep, which provides some reference for the present study and may be worthwhile to be included in future studies for comparative analyses (Zheng, 2019).

#### 4.1.2 The moderating effects of exercise intervention variables

Motor skills can be divided into open and closed skills based on the predictability of environmental changes (Shi and Feng, 2022). Environmental changes in open skills are unpredictable, and practice needs to change with the external environment. Basketball and badminton are exercises in which open skills dominate. For open-skill exercises, the response speed to external stimuli is the key to success or failure. When practicing open-skill exercise, mobile phone addicts are often in a natural, pleasurable exercise environment that facilitates transferring and venting negative emotions. Closed-skill exercises are predictable, and practice is performed constantly; running and swimming are examples of closed-skill exercises (Shi and Feng, 2022). Practicing a closed-skill program can help practitioners focus and strengthen the concentration of mobile phone addicts, thus reducing dependence on the outside world. The mixed skills in this study refer to literature in which both open and closed skills were included in the exercise modalities used. A 2015 survey found that mobile phone addiction reduces the time college students spend on physical activity and that exercise promotes physical health (Kim et al., 2015).

Moreover, exercise increases body temperature, reduces anxiety, and improves the treatment of addictive disorders (Murphy et al., 1996). Meta-analyses depicted that both open and closed skills and mixed skills significantly improved subject total scores on the mobile phone addiction scale, with mixed skills having the largest effect on mobile phone addiction, followed by open and closed skills. All three motor skill interventions had a positive effect on reducing adolescent mobile phone addiction (*p* < 0.05), indicating the effectiveness of different exercise interventions.

Furthermore, the intervention cycle was divided into ≤8 weeks and >8 weeks, with an intervention cycle of 8 weeks or more producing the largest effect. The analysis demonstrated that an exercise cycle that is too short may not achieve the desired intervention effect. Research has shown that both exercise and mobile phone use activate similar neurological pathways in the brain and that long-term exercise can help reduce mobile phone addiction (Kim, 2013). Regarding intervention frequency, the effect size of exercise frequency ≥ 3 times/week was more significant than that of two times/week, and this frequency of exercise is in line with the guidelines of the American College of Sports Medicine (Garber et al., 2011). The higher the frequency of exercise, the higher the likelihood of obtaining exercise benefits. This may be because high-frequency exercise reduces the overall sedentary time and mobile phone use of adolescents and reduces the frequency of mobile phone use, thus reducing mobile phone addiction. Regarding the intervention duration, the most significant effect sizes were found when the duration of single exercise interventions (main exercise time) was 30–60 min, with an average heart rate of 135–150, corresponding to moderate-intensity exercise. High-intensity exercise puts stress on the heart, which induces myocardial injury (Rocha et al., 2012), whereas low-intensity exercise often fails to achieve the desired intervention. Moderate-intensity exercise can effectively improve mitochondrial function and increase myocardial energy metabolism (Veeranki et al., 2016). Typically, 30–60 min of physical activity positively affects the recovery of many addicted patients (Xu et al., 2022). For example, 30 min of Tai Chi exercise was revealed to increase dopamine concentrations and dopamine receptors, which had a positive effect on reducing drug addiction (Wang et al., 2022); 30 min of moderate-intensity exercise was effective in reducing mobile phone cravings among college students with mobile phone addiction (Yang et al., 2022), providing support for our findings. As adolescents are at a stage of rapid physical development, a short exercise session may lack sufficient stimulation, resulting in a lack of enjoyment and a decrease in motivation to participate in exercise; longer exercise sessions, influenced by group exercise, promote interpersonal communication and allow individuals to integrate well into the group. Therefore, choosing a single 30–60 min exercise intervention is more appropriate.

### 4.2 Significance

Many studies have investigated the relationship between exercise and mobile phone addiction; however, studies focusing on the effect of exercise on reducing mobile phone addiction are lacking. This study is the first meta-analysis to focus on the effect of exercise on mobile phone addiction, in which all the included studies were RCTs. Moreover, we conducted subgroup analyses of variables that may have affected the results, considering the heterogeneity sources whenever possible. This study provides strong evidence that exercise ameliorates mobile phone addiction and is of great significance. First, our study confirms that exercise is an effective way to reduce mobile phone addiction in adolescents and further suggests a more comprehensive and effective intervention intensity, frequency, and intervention period from the perspective of exercise prescription. Second, our study concluded that three types of exercise interventions were effective in reducing mobile phone addiction, with mixed skills being the most effective, followed by open skills and closed skills. This finding suggests that a combination of different exercise programs could be a promising exercise protocol to more effectively reduce mobile phone addiction.

### 4.3 Shortcoming and future directions

Despite the significance of this study, it has some limitations. First, our study focused on mobile phone addiction among adolescents, and further validation is required to generalize our findings to other populations. In future studies, attention should be paid to the mobile phone addiction behaviors of different populations, a multidimensional understanding of the effects of exercise interventions on the addiction problems of various groups in society, and targeting to improve the health and quality of life of the target population. Second, even though we reviewed the literature on six databases, only 12 studies and 861 subjects were included, most of which were from China. This suggests that in the future, more rigorous, comprehensive, and high-quality RCTs with different cultural backgrounds should be conducted to provide a reliable theoretical basis for updating research in this area. In addition, neuroscience tools, such as electroencephalogram and transcranial magnetic stimulation, can be considered to investigate the effect of exercise on mobile phone addiction to better address the underlying mechanisms.

## 5 Conclusion

Exercise interventions can reduce mobile phone addiction among adolescents, with varying moderating effects. Based on the findings of this study, we encourage using mixed skills with a duration of 30–60 min for single exercise interventions, three or more exercises weekly, and more than 8 weeks of continuous exercise for adolescents with mobile phone addiction. Additionally, the literature quality and quantity can affect the experimental intervention effect; therefore, it is recommended to follow the experimental specifications to include more studies of higher quality and quantity to explore the improvement effect of exercise interventions on adolescent mobile phone addiction to obtain better intervention effects.

## Author contributions

ZL: Conceptualization, Formal Analysis, Investigation, Methodology, Software, & Writing – review & original draft. XX: Resources, Funding acquisition, Methodology, Writing – review & editing. QS: Software, Validation, Visualization, Writing – review & editing. YL: Conceptualization, Data curation, Supervision, Funding acquisition, Project administration, Writing – review & editing.
